# Prevalence and Genotypes of Mycobacteria Species in Formalin-Fixed Paraffin-Embedded Tissue Samples in a Tertiary Laboratory, Northern Pretoria, South Africa

**DOI:** 10.3390/diagnostics16142234

**Published:** 2026-07-17

**Authors:** Moshawa C. Khaba, Lubabalo Ndam, Ezintle Mhlaba, Lucia N. Mhlongo, Ndivhuho A. Makhado

**Affiliations:** 1 Department of Anatomical Pathology, Dr George Mukhari Academic Laboratory, National Health Laboratory Service, Ga-Rankuwa 0208, South Africa; 2 Department of Anatomical Pathology, Sefako Makgatho Health Sciences University, Pretoria 0204, South Africa; 3 Department of Microbiological Pathology, Sefako Makgatho Health Sciences University, Pretoria 0204, South Africa; 4 Department of Medical Microbiology, Dr George Mukhari Academic Laboratory, National Health Laboratory Services, Ga-Rankuwa 0208, South Africa

**Keywords:** mycobacteria, granulomatous inflammation, *Mycobacterium tuberculosis*, Ziehl–Neelsen, non-tuberculous mycobacteria, Mycobacterium CM/AS assays

## Abstract

**Background/Objectives**: Granulomatous inflammation in tissue biopsies presents a diagnostic challenge, particularly in differentiating between *Mycobacterium tuberculosis* complex (MTB) and non-tuberculous mycobacteria (NTM) in high-HIV-prevalence areas. This study evaluates the prevalence and species distribution of mycobacteria in formalin-fixed, paraffin-embedded (FFPE) tissues from patients with granulomatous inflammation and correlates these findings with demographic and clinical data. **Methods**: Conducted at the NHLS-Dr George Mukhari Tertiary Laboratory, the retrospective descriptive study analysed 37 FFPE samples (January 2019–December 2020) after obtaining ethical approval and collecting clinicopathological data. Histopathological assessments using H&E and Ziehl–Neelsen (ZN) stains were carried out, and mycobacterial species were identified utilising multiplex PCR with reverse hybridisation (GenoType MTBC/CM/AS assays). **Results**: The cohort had an average age of 38.59 years, with a predominance of females (59.5%) and a high HIV positivity rate (62.2%). The most common biopsy site was lymph nodes (32.4%). All samples exhibited necrotising granulomas, but only 8.1% displayed acid-fast bacilli (AFB) on ZN staining. Molecular testing revealed mycobacterial DNA in 75.7% of samples, identifying NTM alone in 50.0%, MTB alone in 10.7%, and both in 28.6%. *M. fortuitum* was the most frequently recognised species. Importantly, there was no significant association between mycobacterial detection and HIV status (*p* > 0.05). **Conclusions**: The findings demonstrate that molecular methods substantially improve mycobacterial detection rates and speciation. This underscores the diagnostic limitations of ZN staining and highlights the importance of molecular diagnostics for accurately identifying bacterial aetiology, crucial for appropriate antimicrobial therapy in immunocompromised patients.

## 1. Introduction

The genus Mycobacterium comprises acid-fast, Gram-positive bacilli that are clinically important and of great public health significance. This genus includes major human pathogens, such as the *Mycobacterium tuberculosis* complex (MTBC), which causes tuberculosis, and *Mycobacterium leprae*, as well as many non-tuberculous mycobacteria (NTM) [1,2]. Tuberculosis (TB) is still the leading infectious disease killer in the world; according to estimates by the World Health Organisation, one-quarter of people on earth are infected with MTBC; more than 10 million active cases have been reported in 2023, along with 1.2 million deaths [3]. Though effective treatment regimens are available, control of the disease can only be achieved if rapid and accurate diagnosis is possible, so that therapy can start early, transmission can be interrupted, and drug resistance will not have time to develop [1,3].

Diagnosis of pulmonary TB has shifted from reliance on culture and smear microscopy to advanced molecular testing. On the other hand, histological diagnosis based on formalin-fixed, paraffin-embedded tissue samples, most often the specimen type encountered in extrapulmonary or paucibacillary disease, has not advanced much beyond the demonstration of acid-fast bacilli (AFB) by Ziehl–Neelsen staining [4]. This method suffers from critically low sensitivity and cannot differentiate MTBC from NTM species, which is critical for life-saving therapy selection [5,6]. When granulomatous inflammation is seen histologically, although mycobacterial disease may be suggested by the presence or absence of AFBs, speciation of the organism is not possible. This led to increased costs, diagnostic delays, and poor guidance for clinicians regarding targeted therapy [6].

This particular diagnostic gap becomes even more important in high-burden resource-limited settings such as South Africa, where HIV co-infection further complicates the spectrum of mycobacterial diseases [7]. Comprehensive studies profiling both MTBC and NTM prevalence and species distribution from these routinely archived FFPE tissues across the African continent are lacking. Thus, a molecular approach was adopted in this study to investigate mycobacterial infections among stored FFPE tissues. The GenoType Mycobacterium CM/AS line probe assay was used to determine the prevalence and diversity of mycobacterial species within histologically identified granulomatous lesions, enabling assessment of this more sensitive and specific diagnostic pathway for tissue-based diagnosis.

## 2. Materials and Methods

The study was a retrospective descriptive study conducted at the National Health Laboratory Service (NHLS), Dr George Mukhari Tertiary Laboratory (DGMTL), in the Department of Anatomical Pathology.

Stored FFPE tissue samples from different organs diagnosed with granulomatous inflammation were retrieved and used in the study between 1 January 2019 and 31 December 2020. Permission for data and facility usage was obtained from the NHLS Academic Affairs and Research Management System (AARMS). The NHLS central data warehouse (CDW) retrieved the cases from the main laboratory system data warehouse using the Systematised Nomenclature of Medicine Clinical Terms (SNOMED) code and word search. The demographic and clinical data, such as age, sex, HIV status and site of biopsy, were collected. This study was granted ethical approval by the Sefako Makgatho Health Science University Research Ethics Committee (SMUREC/M/35/2021). The study followed all ethical guidelines for human research.

### 2.1. Histopathological Review

The 4 µm thick haematoxylin and eosin (H&E)-stained slides were reviewed to evaluate the following morphological features: granulomatous inflammation, presence or absence of necrosis, multinucleated giant cells, and presence or absence of infective organism. Where necessary, recuts at 4 µm were done from archived formalin-fixed paraffin-embedded (FFPE) tissue blocks. Restaining for H&E from archival FFPE blocks was performed according to standard operating procedures (SOPs) for cases in which the H&E slides could not be retrieved or were broken and faded.

### 2.2. Histochemical Stain Review

Archived Ziehl–Neelsen (ZN)-stained slides, used to establish the initial diagnosis by the primary pathologist, were reviewed. The ZN-stained slides were reported for the presence or absence of acid-fast bacilli (AFB). The presence of AFB was further reported as paucibacillary or multi-bacillary. Restaining for ZN from archival FFPE blocks was performed according to standard operating procedures (SOPs) for cases in which the ZN slides could not be retrieved or were broken and faded.

### 2.3. DNA Extraction

The FFPE tissue blocks were retrieved, and 5 ribbons of 10 µm tissue were cut with a sterile surgical blade and placed onto an Eppendorf tube using a sterile applicator stick. To reduce cross-contamination, a sterile blade was used, and the microtome was cleaned with xylene and absolute ethanol (100%). Kleenex tissue and Sellotape were used and changed for each tissue sample. The tissues were then deparaffinised in xylene, rehydrated in decreasing alcohol grades (100%, 95%, 85%, and 70% ethanol), and rinsed in sterile distilled water.

Tissue sections were placed into 1.5 mL microcentrifuge extraction tubes. A total of 1 mL of xylene was added to the microcentrifuge tubes, and the samples were vortexed to remove paraffin. The suspension was incubated at room temperature for 60 min with gentle rocking, then centrifuged at 10,000× *g* for 5 min. The supernatant was discarded, and the step was repeated. A total of 1 mL of 100%, 95%, and 75% ethanol was used to wash the tissue sample twice for 5 min each, followed by rinsing with 1 mL of sterile double-distilled water for 5 min, then vortexing. The supernatant was discarded.

A total of 45 µL sterile water, 45 µL of 2× digestion buffer and 10 µL Proteinase K were added to the deparaffinised tissue sample in a microcentrifuge tube and incubated at 55 °C for 4 h. The suspension was transferred onto a 94 °C heating block and incubated for 20 min. About 5 μL of RNase A was added, vortexed and incubated for a further 5 min at room temperature. A total of 350 μL of genomic lysis buffer was added to the tube, and the tube was vortexed. The supernatant was transferred into a Zymo-Spin™ IICR Column (Zymo Research Corporation, Irvine, CA, USA) in a collection tube and centrifuged at 10,000× *g* for 1 min. About 400 μL of Genomic DNA Wash 1 was added to the spin column in a new collection tube, and the mixture was centrifuged at 10,000× *g* for 1 min. The flow-through was discarded. This was followed by adding 700 μL of Genomic DNA Wash 2 to the spin column and centrifuging at ≥12,000× *g* for 1 min. The flow-through was discarded. A total of 200 μL of Genomic DNA Wash 2 was added to the spin column. This was centrifuged at ≥12,000× g for 1 min. Finally, the Zymo-Spin™ IICR Column was transferred to a clean microcentrifuge tube, and ≥50 μL DNA elution buffer was added to the spin column. This was incubated for 2–5 min at room temperature and centrifuged at top speed for 30 s to elute the genomic DNA. The eluted DNA was stored at −20 °C for future use.

### 2.4. DNA Amplification

Multiplex PCR was performed in a 50 µL reaction mixture containing 5 µL of extracted DNA, 35 µL of primer–nucleotide mix, 5 µL of 10× polymerase incubation buffer, MgCl_2_, 1–2 unit(s) of thermostable DNA polymerase, and molecular-grade water. The PCR master mix was prepared in a separate room free of contaminating DNA. Amplification was carried out as follows: an initial denaturation hold cycle at 95 °C for 15 min, followed by 10 cycles of annealing and extension at 95 °C for 30 s and 65 °C for 2 min, 20 cycles at 95 °C for 25 s, 50 °C for 40 s, and 70 °C for 40 s, and the lastly 1 cycle of extension at 70 °C for 8 min. The amplicons were stored at −20° C for further use.

### 2.5. Reverse Hybridisation and Detection

Hybridisation was conducted on the automated GT blot 48 Hain Life Science-Bruker, Nehren, Germany, following the manufacturer’s instructions. Briefly, 20 µL of denaturation buffer was thoroughly mixed with 20 µL of the amplified sample in a plastic 48-well tray and incubated at room temperature for 5 min. Subsequently, hybridisation was performed for 30 min at 45 °C, followed by a stringent wash of unbound amplicons for 15 min at 45 °C. After aspirating the solution, the strips were rinsed with a rinsing solution and washed with sterile ddH_2_O. Then, 1 mL of conjugate buffer was added to each strip, and the strips were incubated for 30 min at room temperature. The solution was discarded, rinsed twice with the rinse buffer, and then washed with sterile ddH_2_O. A total of 1 mL of substrate buffer was added to each strip, and the strips were incubated for 5 to 8 min at room temperature. All solutions were removed, and the reaction was stopped by two rinses with ddH_2_O. The test strips were dried, and the results were scanned and interpreted using a GenoScan (Hain LifeScience, Bruker, Nehren, Germany) according to the manufacturer’s instructions.

Quality control strains processed with each test run including MTB H37Rv ATCC 25177 and *M. kansasii* ATCC 12478 were used as positive controls and sterile double-distilled water (ddH_2_O) as a negative control.

### 2.6. Statistical Analysis

Raw data on participants’ profiles were captured in a Microsoft Excel spreadsheet (Microsoft Office 2016) and cleaned before further analysis. The data were then exported to STATA v.18 (StataCorp, College Station, TX, USA) for analysis. Descriptive data analysis was performed by calculating frequencies, proportions, and percentages for categorical variables and the mean for continuous variables such as age. Percentages were used to present the prevalence of the different mycobacterial species in the study. The Chi-square test was used to analyse the association between mycobacterial infections and demographic data. A *p*-value less than 0.05 was considered statistically significant for this study.

## 3. Results

### 3.1. Demographics

The study cohort comprised 37 cases of granulomatous inflammation. Females constituted the majority of cases, accounting for 59.5% (*n* = 22), while males accounted for 40.5% (*n* = 15) of the study population. This indicates a slight female predominance among the cases analysed in this study.

**Figure 1:**
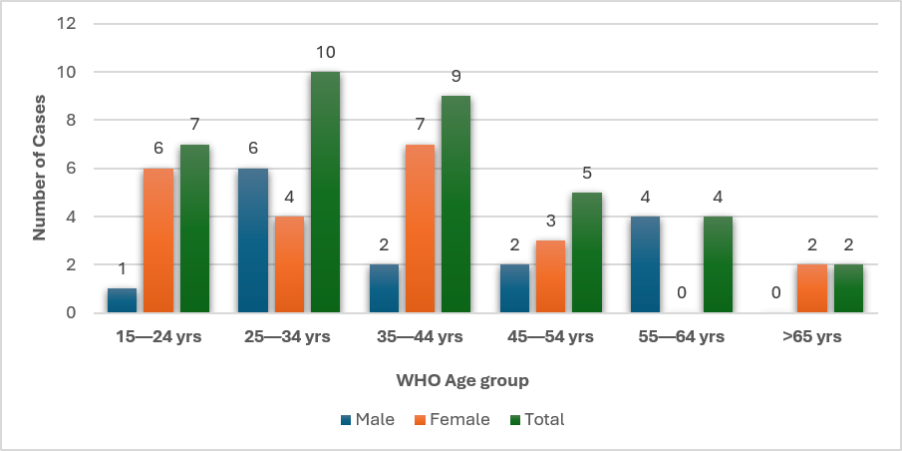
Age and gender distribution of the study cases as per the WHO age group.

The average age was 38.59 ± 15.43 years old (range 17–83 years). The patients who were aged less than 40 years (<40 years) were the most affected by mycobacterial organisms, 59.5% (*n* = 22) (Figure 1).

### 3.2. HIV Status

**Figure 2:**
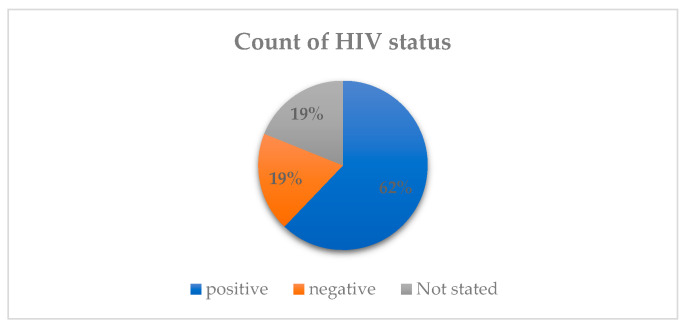
The HIV status of the participants.

HIV status was available for the majority of the study participants. Among the 37 cases, 62.2% (23/37) were HIV-positive, while 18.9% (7/37) were HIV-negative. In the remaining 18.9% (7/37) of cases, HIV status was not documented in the available clinical records. The information on CD4 count, viral load, and antiretroviral therapy (ART) was inaccessible. The distribution of HIV status among the study population is illustrated in Figure 2.

### 3.3. Biopsy Site

**Figure 3:**
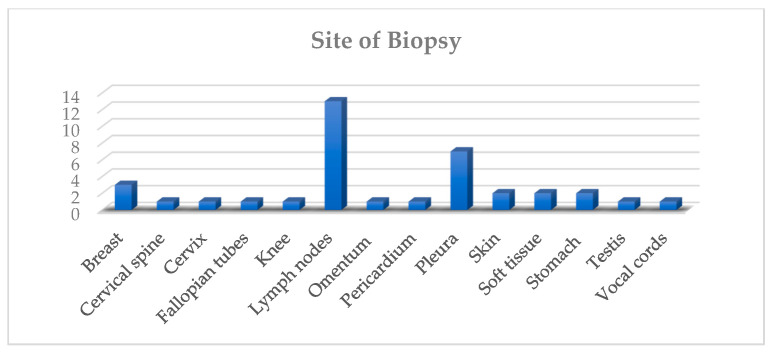
Illustrates the site distribution of the tissue specimens.

The distribution of biopsy sites among the study cases was diverse, reflecting the varied anatomical involvement of granulomatous disease. Lymph nodes were the most common biopsy site, accounting for 35.1% (*n* = 13) of cases. This was followed by the pleural site, which comprised 18.9% (*n* = 7) of cases. Breast origin accounted for 8.1% (*n* = 3) of biopsies, while soft tissue, stomach, and skin each accounted for 5.4% (*n* = 3). The remaining sites, including cervical spine, cervix, fallopian tube, knee, omentum, pericardium, testis, and vocal cords, were each represented by a single case (2.7%). The distribution of biopsy sites is illustrated in Figure 3.

### 3.4. Pathology

**Figure 4:**
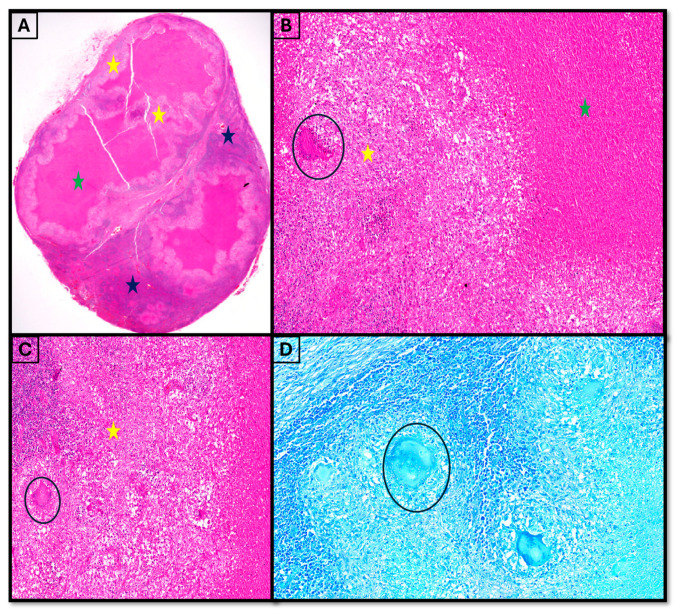
Micrographs illustrate histological features of the lymph node with necrotising granulomatous inflammation (**A**–**C**), (**A**) ×4 magnification; (**B**,**C**) ×10 magnification. (**D**) ×20 magnification showing absent acid-fast bacilli on ZN stain. Black stars show lymphoid component of lymph node. Yellow stars show epithelioid histiocytes. Green stars show caseative necrosis. Black circles show multinucleated giant cells.

All the cases showed necrotising granulomatous inflammation composed of aggregates of epithelioid histiocytes, a cuff of lymphocytes and multinucleated giant cells with central caseative necrosis. Fungi or parasites were not seen. Of the 37 cases, only 5.4% (*n* = 2) showed acid-fast bacilli on ZN staining, indicating paucibacillary disease. However, Figure 4 shows negative ZN.

### 3.5. Polymerase Chain Reaction Findings

**Figure 5:**
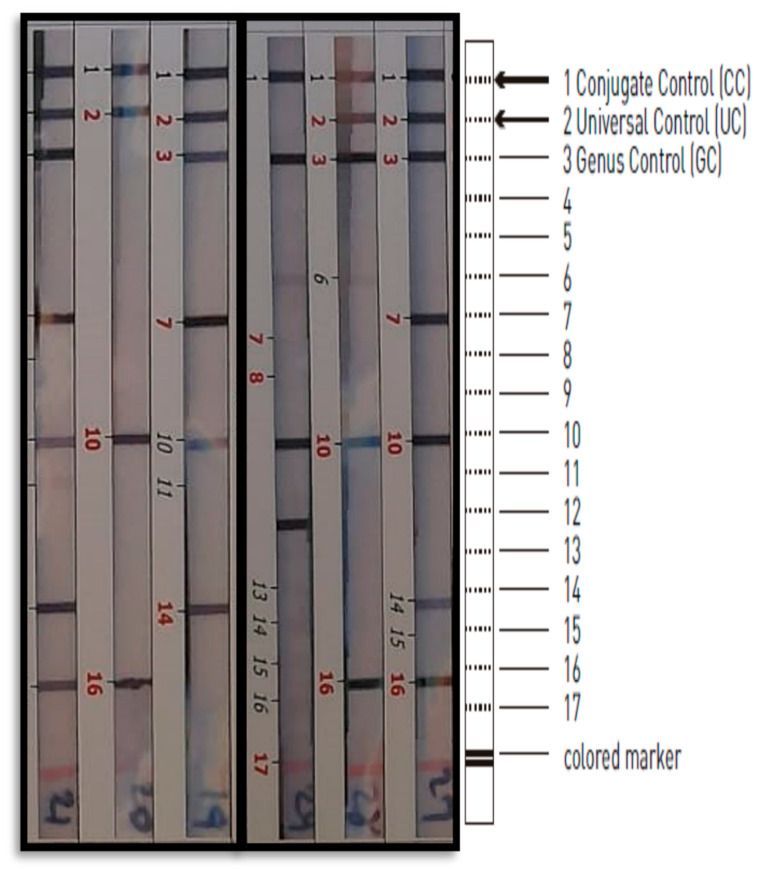
GenoType Mycobacterium CM/AS assay results for rapid identification of *Mycobacterium tuberculosis* complex and different non-tuberculous mycobacteria species following the banding patterns as per the interpretation chart, according to the manufacturer’s instructions. From left to right: strip 1—a mixture of *Mycobacterium fortuitum* and *Mycobacterium tuberculosis* complex; strip 2—*Mycobacterium tuberculosis* complex; strip 3—a mixture of *Mycobacterium tuberculosis* complex and *Mycobacterium* species; strip 4—*Mycobacterium kansasii*, strip 5—*Mycobacterium tuberculosis* complex; strip 6—a mixture of *Mycobacterium fortuitum* and *Mycobacterium tuberculosis* complex.

The overall prevalence of mycobacterial infection in these tissue samples was 75.7% (*n* = 28). Mycobacterial DNA was not detected in 24.3% (*n* = 9) of the analysed tissue samples (Figure 5).

### 3.6. Mycobacteria Species

**Figure 6:**
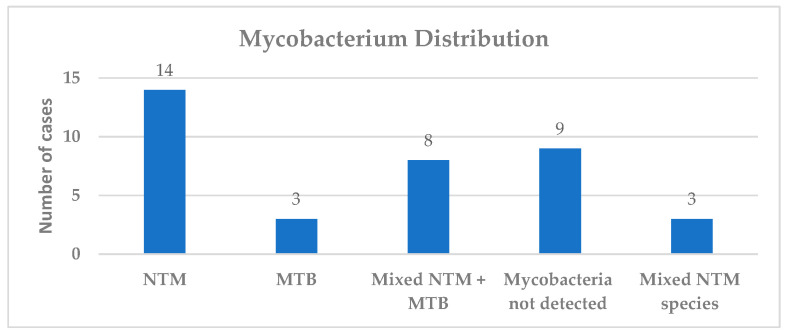
Distribution of mycobacterial species detected by molecular analysis in FFPE tissue samples. *M. fortuitum* and mixed *M. fortuitum*–MTB infections were the most frequently identified organisms.

Molecular characterisation further demonstrated a predominance of non-tuberculous mycobacteria (NTM) in the study cohort. Among the PCR-positive cases, NTM species were identified in 50.0% (*n* = 14), while mixed infections involving both NTM and *Mycobacterium tuberculosis* (MTB) complex were detected in 28.6% (*n* = 8). MTB complex alone was identified in 10.7% (*n* = 3) of cases. In addition, mixed infections involving multiple NTM species accounted for 10.7% (*n* = 3) of PCR-positive samples. The distribution of the identified mycobacterial species and infection categories by biopsy site is illustrated in Figure 5 and Figure 6 and Table 1.

### 3.7. Comparison of ZN and PCR

Molecular testing detected mycobacterial DNA in 75.7% (*n* = 28) of cases, compared with only 8.1% (*n* = 3) detected by Ziehl–Neelsen staining. The difference in detection rates between PCR and Ziehl–Neelsen staining was statistically significant (*p* < 0.001, McNemar test), indicating that PCR detected significantly more mycobacterial infections than Ziehl–Neelsen staining (Table 2).

### 3.8. Mycobacteria Species and HIV Status

**Figure 7:**
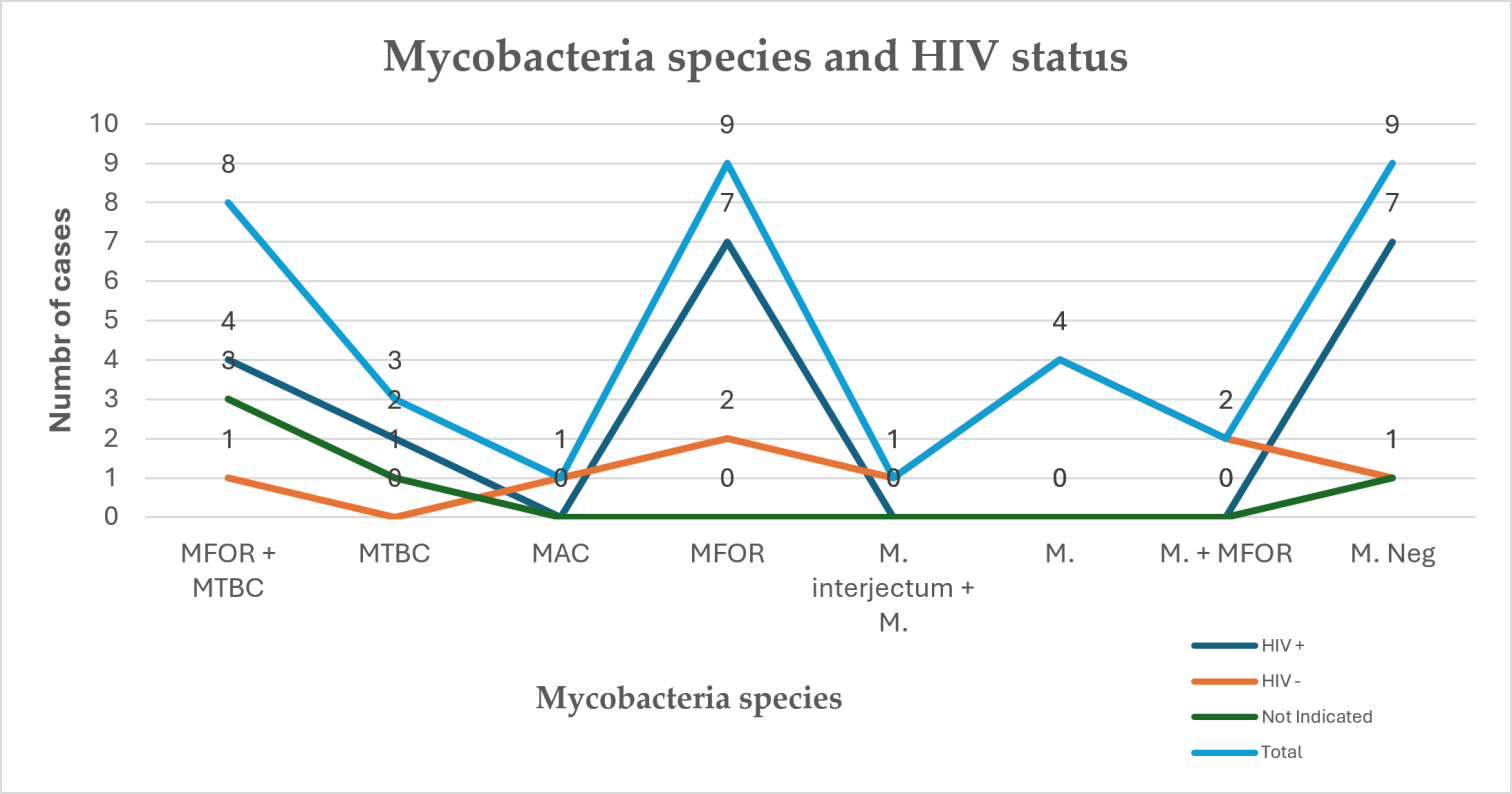
The trends of mycobacteria species detected on formalin-fixed paraffin-embedded tissue samples associated with HIV status. MFOR: *Mycobacterium fortuitum*, MTBC: *Mycobacterium tuberculosis* complex, MAC: *Mycobacterium avium complex*, M. interjectum: *Mycobacterium interjectum*, M.: *Mycobacterium* spp., M. neg: *Mycobacterium* negative.

*M. fortuitum* and both MTB complex and *M. fortuitum* were the most frequently identified mycobacterial organisms in this study for the HIV-positive, HIV-negative, and the group with HIV status not indicated. M. avium was the least detected and was detected in the HIV-positive group (Figure 7). No statistically significant association was observed between HIV status and molecular detection of mycobacteria (*p* = 0.64) using Fisher’s exact test (Table 3).

## 4. Discussion

This study demonstrated that PCR-based molecular testing substantially improved the detection of mycobacterial infection in archived FFPE tissue compared with ZN staining. In addition to identifying MTBC, PCR detected several NTM, highlighting the diagnostic value of molecular methods in evaluating granulomatous inflammation, particularly in high TB- and HIV-burden settings.

The female predominance observed in this study was contrary to some global reports, which often show a higher burden in males; however, this may reflect gender-specific healthcare-seeking behaviour or regional epidemiological patterns of cases [3]. Importantly, the HIV-positivity in this study highlights the persistent and profound association of HIV infection and mycobacterial disease. HIV impairs cell-mediated immunity, which significantly increases the risk of severe and disseminated disease [3,8].

The most frequent biopsy site was the lymph node, consistent with the high incidence of extrapulmonary disease in immunocompromised patients. Diagnosis at extrapulmonary sites is difficult due to a lower mycobacterial burden and reduced sensitivity of conventional methods compared with those used for pulmonary disease [9,10,11]. The high PCR positivity across biopsy sites in this study underscores the utility of molecular testing in the workup of extrapulmonary granulomatous disease [12,13].

Necrotising granulomatous inflammation was seen in all cases; this feature is not specific to MTBC and cannot distinguish tuberculosis from NTM infection. The low positivity rate of ZN staining reflects the limited sensitivity in paucibacillary disease. Negative histochemical findings do not exclude mycobacterial infection [5,6]. In fact, ZN staining cannot detect mycobacteria below about 10,000 organisms/mL, making it inherently insensitive in paucibacillary disease, such as that seen in extrapulmonary infections and immunocompromised patients [14].

This underscores the importance of ancillary molecular testing, especially PCR on FFPE tissue, in the setting of necrotising granulomas with negative ZN stains. PCR played a major role in speciation and significantly increased the detection rates, guiding appropriate therapy [6]. One of the key findings was that the better diagnostic performance of PCR than ZN staining is that PCR was able to detect mycobacterial DNA in specimens with a low bacillary load by amplifying small amounts of nucleic acid below the threshold of microscopic detection, which is in line with previous studies that have demonstrated greater sensitivity of molecular methods than conventional methods [6,13,14].

In the current study, PCR markedly improved the detection and identification of mycobacteria compared with ZN staining and identified clinically relevant NTM that were not detected by histopathology alone. These results support the application of PCR in diagnostic workups when histopathology and special stains are inconclusive, especially in high-burden, resource-limited settings [15].

Species identification also revealed the presence of clinically important NTM, particularly *M. fortuitum*, as well as mixed infections with MTBC. The prominence of NTM, particularly *M. fortuitum*, may signal a shift in the epidemiological trends of mycobacterial infection, requiring a high clinical index of suspicion and laboratory capacity for definitive species identification, as treatment regimens for NTM differ drastically from those for MTB [15,16]. Failure to distinguish MTBC from NTM may therefore result in inappropriate treatment and poorer clinical outcomes [16,17].

The genotypic heterogeneity was greatest in lymph nodes, with *M. avium*, *M. intracellulare*, and mixed *M. fortuitum*/MTBC patterns consistent with a common drainage site for diverse mycobacterial infections. *M. fortuitum* alone or in combination with MTBC complex was common at extrapulmonary and unusual sites (pleura, breast, stomach, vocal cords, cervical spine, fallopian tubes), suggesting this fast-growing environmental species can coexist with or mimic tuberculous disease. Co-infections are uncommon but are increasingly reported in immunocompromised hosts [18,19]; deep sequencing can reliably differentiate true co-infection from contamination [20,21] and should be used for further confirmation.

Most patients were HIV-positive, but there was no statistically significant association between HIV status and PCR positivity. This finding should be interpreted with caution, given the small sample size and the absence of key clinical variables, including CD4 cell count, HIV viral load, and antiretroviral therapy status [6]. These variables are important indicators of immune function and disease control, and may influence susceptibility to mycobacterial infection and the spectrum of infecting species, especially opportunistic NTM [9]. Because they were absent, it was not possible to determine whether the observed detection rates and species distributions reflected the degree of immunosuppression rather than just HIV status [7]. Further characterisation of the relationship between HIV-associated immunosuppression and molecular detection of mycobacterial disease requires larger prospective studies with complete clinical and immunological data.

The principal strength of this study is the application of PCR to routinely processed FFPE tissue, demonstrating the feasibility of identifying molecular species in archived pathological specimens. However, the retrospective design, relatively small sample size, and incomplete clinical information limited subgroup analyses, while DNA degradation in archived tissue may have influenced PCR performance. Although the use of a negative control sample was included from the gDNA extraction step until the detection step, this study is limited by the lack of a negative biopsy from a healthy individual to rule out the possibility of picking up colonising NTM. Similarly, it offers crucial insight into improving NTM diagnosis from the widely and readily available FFPE tissues.

## 5. Conclusions

The results of the present study provide strong evidence that PCR-based molecular testing is more sensitive than ZN staining for detecting mycobacterial infections in FFPE tissue. The predominance of NTM species in this cohort highlights the importance of molecular speciation in the diagnostic workup, as it has direct implications for clinical management. These findings provide strong support for the wider implementation of molecular diagnostics into the practice of anatomical pathology in settings of high mycobacterial disease burden.

## Figures and Tables

**Table 1:** Distribution of genotypes according to site of biopsy.

Site of Biopsy	Genotype/Species Identified	*n*
Lymph node (*n* = 9)	*M. avium*	1
	*M. interjectum* and *Mycobacterium* spp.	1
	*M. fortuitum* and Mtb complex	3
	Mtb complex	2
	*Mycobacterium* spp.	2
Pleura (*n* = 4)	Mtb complex	1
	*M. fortuitum*	3
Breast (*n* = 3)	*M. fortuitum* and Mtb complex	2
	*M. fortuitum*	1
Stomach (*n* = 2)	*M. fortuitum*	2
Knee (*n* = 1)	*Mycobacterium* spp. and *M. fortuitum*	1
Muscle of hip and thigh (*n* = 1)	*Mycobacterium* spp. and *M. fortuitum*	1
Submental region (*n* = 1)	*M. fortuitum* and Mtb complex	1
Soft tissue (*n* = 1)	*M. fortuitum* and Mtb complex	1
Testis (*n* = 1)	*M. fortuitum* and Mtb complex	1
Skin (*n* = 1)	*Mycobacterium* spp.	1
Omentum (*n* = 1)	*Mycobacterium* spp.	1
Vocal cords (*n* = 1)	*M. fortuitum*	1
Cervical spine (*n* = 1)	*M. fortuitum*	1
Fallopian tubes (*n* = 1)	*M. fortuitum*	1
Total		28

**Table 2:** Comparison of Ziehl–Neelsen staining and PCR detection.

Diagnostic Method	Positive *n* (%)	Negative *n* (%)	Significance
Ziehl–Neelsen	3 (8.1%)	34 (91.9%)	*p* < 0.001
PCR detection	28 (75.7%)	9 (24.3%)	

**Table 3:** Association between HIV status and mycobacterial detection.

HIV Status	PCR Positive	PCR Negative	Total	Significance
Positive	16	7	23	*p* = 0.64
Negative	6	1	7	
Not stated	6	1	7	

## Data Availability

The original contributions presented in this study are included in the article. Further inquiries can be directed to the corresponding author.

## References

[1] Gordon S.V., Parish T. (2018). Microbe profile: *Mycobacterium tuberculosis*: Humanity’s deadly microbial foe. Microbiology.

[2] Petrini B. (2006). Non-tuberculous mycobacterial infections. Scand. J. Infect. Dis..

[3] World Health Organization (2023). Global Tuberculosis Report 2023.

[4] Kwon Y.S., Koh W.J. (2016). Diagnosis and treatment of nontuberculous mycobacterial lung disease. J. Korean Med. Sci..

[5] Cho M.S., Lee S.N., Sung S.H., Han W.S. (2003). Comparison of Ziehl-Neelsen stain and TB-PCR on detection of *Mycobacterium tuberculosis* in formalin-fixed, paraffin-embedded tissues of chronic granulomatous inflammation. Korean J. Pathol..

[6] Lee H.S., Park K.U., Park J.O., Chang H.E., Song J., Choe G. (2011). Rapid, sensitive, and specific detection of *Mycobacterium tuberculosis* complex by real-time PCR on paraffin-embedded human tissues. J. Mol. Diagn..

[7] Dawood H., Richards L., Lutchminarain K., Parker A., Wattrus C., Sipambo N., Nel J., Manzini T., Naidoo K. (2024). Southern African HIV Clinicians Society guideline on the management of non-tuberculous mycobacteria in people with HIV. S. Afr. J. HIV Med..

[8] Kwan C., Ernst J.D. (2011). HIV and tuberculosis: A deadly human syndemic. Clin. Microbiol. Rev..

[9] Chiang C.H., Tang P.U., Lee G.H., Chiang T.H., Chiang C.H., Ma K.S.K., Fang C.T. (2021). Prevalence of nontuberculous mycobacterium infections versus tuberculosis among autopsied HIV patients in Sub-Saharan Africa: A systematic review and meta-analysis. Am. J. Trop. Med. Hyg..

[10] Meghdadi H., Khosravi A.D., Ghadiri A.A., Sina A.H., Alami A. (2015). Detection of *Mycobacterium tuberculosis* in extrapulmonary biopsy samples using PCR targeting IS6110, rpoB, and nested-rpoB PCR Cloning. Front. Microbiol..

[11] Tortoli E. (2009). Clinical manifestations of nontuberculous mycobacteria infections. Clin. Microbiol. Infect..

[12] Negi S.S., Singh P., Sharma K. (2023). Molecular diagnosis of tuberculosis. Diagnosis of Mycobacterium.

[13] Ulrichs T., Lefmann M., Reich M., Morawietz L., Roth A., Brinkmann V., Kosmiadi G.A., Seiler P., Aichele P., Hahn H. (2005). Modified immunohistological staining allows detection of Ziehl-Neelsen-negative *Mycobacterium tuberculosis* organisms and their precise localization in human tissue. J. Pathol..

[14] Mehta P.K., Raj A., Singh N., Khuller G.K. (2012). Diagnosis of extrapulmonary tuberculosis by PCR. FEMS Immunol. Med. Microbiol..

[15] Daley C.L., Iaccarino J.M., Lange C., Cambau E., Wallace R.J., Andrejak C., Böttger E.C., Brozek J., Griffith D.E., Guglielmetti L. (2020). Treatment of nontuberculous mycobacterial pulmonary disease: An official ATS/ERS/ESCMID/IDSA clinical practice guideline. Clin. Infect. Dis..

[16] Koh W.J., Kwon O.J., Lee K.S. (2005). Diagnosis and treatment of nontuberculous mycobacterial pulmonary diseases: A Korean perspective. J. Korean Med. Sci..

[17] Ratnatunga C.N., Lutzky V.P., Kupz A., Doolan D.L., Reid D.W., Field M., Bell S.C., Thomson R.M., Miles J.J. (2020). The Rise of Non-Tuberculosis Mycobacterial Lung Disease. Front. Immunol..

[18] Kham-Ngam I., Chetchotisakd P., Ananta P., Chaimanee P., Sadee P., Reechaipichitkul W., Faksri K. (2018). Epidemiology of and risk factors for extrapulmonary nontuberculous mycobacterial infections in Northeast Thailand. PeerJ.

[19] Lopez P., Ali W., Donaldy W., Raien G.S., Sivapalan V. (2025). Disseminated Extrapulmonary Tuberculosis and Pulmonary *Mycobacterium avium* Complex Co-infection in a Newly Diagnosed HIV Patient: A Case Report. Cureus.

[20] Opperman C., Steyn J., Matthews M.C., Singh S., Ghebrekristos Y., Kerr T.J., Miller M., Esmail A., Cox H., Warren R. (2024). Targeted deep sequencing of mycobacteria species from extrapulmonary sites not identified by routine line probe assays: A retrospective laboratory analysis of stored clinical cultures. IJID Reg..

[21] Okoi C., Anderson S.T.B., Antonio M., Mulwa S.N., Gehre F., Adetifa I.M.O. (2017). Non-tuberculous Mycobacteria isolated from Pulmonary samples in sub-Saharan Africa—A Systematic Review and Meta Analyses. Sci. Rep..

